# Aging-Shifted Prostaglandin Profile in Endothelium as a Factor in Cardiovascular Disorders

**DOI:** 10.1155/2012/121390

**Published:** 2012-02-13

**Authors:** Hao Qian, Na Luo, Yuling Chi

**Affiliations:** Department of Medicine, Albert Einstein College of Medicine, Bronx, NY 10461, USA

## Abstract

Age-associated endothelium dysfunction is a major risk factor for the development of cardiovascular diseases. Endothelium-synthesized prostaglandins and thromboxane are local hormones, which mediate vasodilation and vasoconstriction and critically maintain vascular homeostasis. Accumulating evidence indicates that the age-related changes in endothelial eicosanoids contribute to decline in endothelium function and are associated with pathological dysfunction. In this review we summarize currently available information on aging-shifted prostaglandin profiles in endothelium and how these shifts are associated with cardiovascular disorders, providing one molecular mechanism of age-associated endothelium dysfunction and cardiovascular diseases.

## 1. Introduction

Cardiovascular disorders, including atherosclerosis, coronary artery disease, heart failure, and hypertension, remain the leading cause of death worldwide [1]. These diseases are among several pathological conditions that are associated with aging [2–4], and age is a primary risk factor for their development [5, 6]. Endothelium is a thin layer of epithelial cells which line the interior of lymph and blood vessels and is a major component of the vascular wall. One important contributor to the development of cardiovascular diseases is a dysfunctional endothelium. Endothelial dysfunction is considered a fair predictor of cardiovascular diseases [4, 7–11].

Furchgott and Zawadzki unequivocally demonstrated that the endothelium is required for normal vessel relaxation [12]. Besides inducing relaxation, normal and healthy endothelium regulates vessel wall permeability, blood flow, vascular tone, and structure and exerts anticoagulant and fibrinolytic properties [13]. Aging adversely affects these normal functions of the endothelium, enhancing vasospasm and thrombosis, leading to eventual cardiovascular diseases [4, 14–16]. Age-impaired vascular relaxation has been shown in different human vascular beds including brachial artery, aorta, coronary artery, carotid, and mesenteric microvessels [14–21]. In line with these reports, additional evidence has been obtained in different vascular beds of animals including dogs [2, 22], rats [2, 23–32] and mice [33, 34]. This reduced relaxation is accompanied with increased blood pressure [35–39]. Elevated blood pressure is an important cardiovascular risk factor that can eventually lead to heart failure.

Normal endothelial function is regulated by a controlled balance between endothelium-dependent relaxing factors and endothelium-dependent contracting factors. The main vasoactive factors released by endothelial cells are nitric oxide (NO) and cyclooxygenase- (COX-) derived eicosanoids [4, 40, 41]. NO production has been shown to be reduced with aging [42–45]. There is less information on how eicosanoids change in the endothelium with age. It is also not well understood how changes in eicosanoid profile might contribute to endothelium dysfunction. Nevertheless, accumulating evidence indicates that the age-related changes in endothelial eicosanoids contribute to endothelium dysfunction and to the development of age-associated cardiovascular diseases.

**Figure 1:**
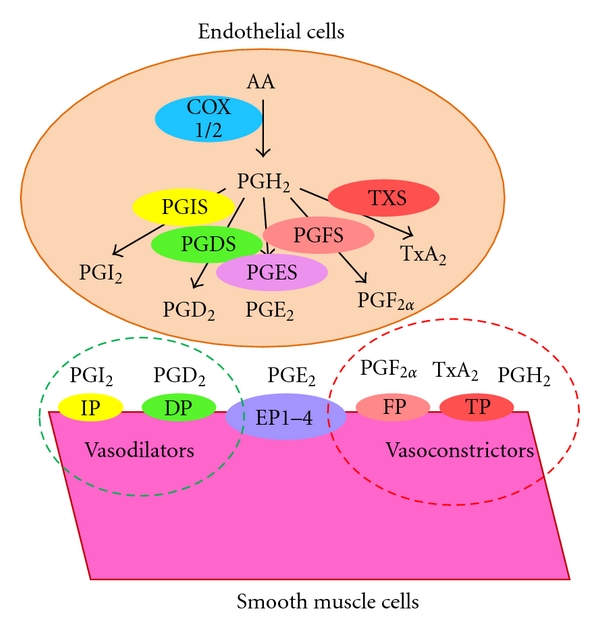
Synthesis and signaling of PGs in the vascular system. Upon stimulation, AA is released from the endothelial cell membrane to the cytosol where it is enzymatically converted to PGH_2_ by COX1 and COX2. Subsequently, PGH_2_ is transformed to PGI_2_, PGE_2_, PGD_2_, PGF_2*α*_, and TxA_2_. These substances, as well as untransformed PGH_2_, are released out of endothelial cells and into the circulation, where they interact with their receptors localized on the smooth muscle cell surface and trigger vasoactive signals.

In endothelium, there are six primary cyclooxygenase-(COX-) derived eicosanoids, prostaglandin H_2_ (PGH_2_), prostaglandin I_2_ (PGI_2_, prostacyclin), prostaglandin E_2_ (PGE_2_), prostaglandin F_2*α*_ (PGF_2*α*_), prostaglandin D_2_ (PGD_2_), and thromboxane A_2_ (TxA_2_) (Figure 1). These eicosanoids are local hormones that are synthesized by virtually all mammalian tissues [46] and act at or near their sites of synthesis in both autocrine and paracrine fashion. They trigger a vast array of biological signals, among which are vasodilation, vasoconstriction, and platelet aggregation [47–49]. In fact, the eicosanoids were the first identified endothelium-derived vasoactive factors [50, 51]. Although there is conflicting evidence [52–54], the majority of the literature shows that PGI_2_ and PGD_2_ are vasodilators [55–59], whereas PGH_2_, PGF_2*α*_, and TxA_2_ are vasoconstrictors and/or platelet aggregation inducers [53, 54, 60–66]. PGE_2_ can induce vasodilation [47, 67–70] or vasoconstriction [53, 54, 71–73], depending on the vascular bed and concentration [74, 75]. In healthy endothelium, these vasodilators and vasoconstrictors, coexisting with other vasoactive factors, are held in balance to maintain normal vascular functions. The aging process shifts this balanced profile toward a proconstrictive mediator profile [76, 77]. In this paper, we summarize and discuss how endothelium-derived eicosanoid profile changes with age and how those changes might contribute to age-associated endothelium dysfunction.

There is limited data on how eicosanoids change in humans [4], and most experiments have been conducted in animal models and most commonly in rat [2]. Rats of 1.5–2 months or less are considered immature, rats of 3–6 months are considered young adult, and rats of approximately 24 months or more are considered aged, though there are differences between strains [2].

## 2. Cyclooxygenases and PGH_2_

There are two isoforms of the cyclooxygenases (COX1 and COX2) encoded by two different genes. Both COX1 and COX2 are expressed in the endothelial and vascular smooth muscle cells, and the expression levels are 20-fold higher in endothelial cells than in smooth muscle cells [78]. In endothelium, both of the COX enzymes are constitutively expressed [79, 80]. However, they are also inducible, for instance, by shear stress [79–81]. Endothelial cells express COX1 preferentially over COX2 [82, 83].

In human mesenteric microvessels of individuals greater than 80 years of age, COX1 levels are 50% increased, while COX2 levels are slightly decreased [21]. In normotensive rats, both COX1 and COX2, in either whole vascular tissue or endothelial cells from vasculatures, are increased with aging from 1-fold to 5-fold [29, 42, 63, 84–86]. Comparable effects of aging on COX1 and COX2 expression levels have been observed in mice [33, 34]. At similar ages, COX1 or COX2 expression, measured at the mRNA or protein levels, is almost doubled in the aorta of spontaneous hypertensive rats (SHRs) as compared to normotensive control Wistar-Kyoto (WKY) rats [63, 84, 87, 88]. Similar increases in COX1 and COX2 were observed in N^*ω*^-nitro-L-arginine methyl ester-(L-NAME-) induced hypertensive rats as compared to control Sprague-Dawley rats [89]. Increased COX2 was also reported in the renal artery of hypertensive patients [89]. These data indicate that there are age-associated increases in COX1 and COX2 levels, as well as an association between elevated COX1/COX2 levels, in both animal models and human studies, and clinical cardiovascular disorders.

Upon stimulation, arachidonic acid (AA) is released from the cell membrane to the cytosol where it is enzymatically converted to PGH_2_ by COX1 and COX2 (also referred to as synthase, PGHS1, and synthase 2, PGHS2, resp.). As shown in Figure 1, PGH_2_ is the common precursor of other prostaglandins and TxA_2_. It is transformed to various PGs and TxA_2_ by a corresponding specific terminal synthase. Besides serving as a common precursor, untransformed PGH_2_ can trigger signals such as vasoconstriction and platelet aggregation by interacting with TxA_2_ receptor (TP) [25, 26, 90–99]. Although no evidence has directly shown the attenuation of untransformed and bioactive PGH_2_ during aging or in cardiovascular pathology, likely due to instability and difficulty in measurement [25, 26, 91, 92, 95, 96], increased COX1 and COX2 associated with aging [21, 29, 42, 63, 84–86] and hypertension [63, 84, 87, 88] would be predicted to result in increases in untransformed PGH_2_. Indirect evidence is provided by reports of reduced vasoconstriction of aortas of aged and/or hypertensive rats by inhibitors of PGH_2_ synthases, rather than TxA_2_ synthase (TXS) [25, 26, 91, 92, 96]. Although the vascular contraction induced by AA has mainly been attributed to TxA_2_ [100, 101], the efficacy of PGHS inhibition, but poor efficacy for a TXS inhibitor, in inducing relief from vasoconstriction provides evidence for PGH_2_ as a vasoconstrictor [91, 92, 96]. 

## 3. PGI_2_

PGI_2_ (prostacyclin) is the first described metabolite of arachidonic acid, and endothelium is the major site of its biosynthesis [51, 57]. In endothelium, both COX1 and COX2 are the upstream contributors of PGI_2_ synthesis [80, 102–104]. PGI_2_ is synthesized by its terminal specific PGI_2_ synthase (PGIS) [105, Figure  1]. PGIS colocalizes with COX1 in endothelial cells [106]. In endothelium, PGIS is by far the most abundant PG terminal synthase, with its expression level 5–100-fold higher than the other PG terminal synthases [54, 64, 65, 84]. Accordingly, PGI_2_ is the most abundant endothelial eicosanoid, with expression levels 10–100-fold higher than that of the other eicosanoids in humans [107, 108] and in animals [54, 97, 109, 110].

PGI_2_ triggers potent vasodilation [51, 57] by interacting with the PGI_2_ receptor (IP) (Figure 1), which located in smooth muscle cells [108, 111]. The vasodilation effect of PGI_2_ has also been shown in pig coronary arteries at low concentrations [58]. At higher concentrations PGI_2_ may induce vasoconstriction [32, 54, 64]. PGI_2_ cannot cause vasoconstriction until its concentration reached 1 *μ*M or higher. 1 *μ*M is 1000-fold higher than the endogenous concentration of PGI_2_, which is in the 0.2–1 nM range [112]. Even at elevated concentrations, PGI_2_ is a weak vasoconstrictor and induces modest tension in the rat aorta [32, 54, 64]. Modest vasoconstrictive effects of PGI_2_ may emanate from weak cross-activation of TP, which can induce vasoconstriction [49]. At lower concentrations, PGI_2_, especially endogenous PGI_2_, is a vasodilator. In addition, PGI_2_ is the most potent endogenous anticoagulation agent [113]. The vasodilation and anticoagulation effects of PGI_2_ have been confirmed by a recent report showing that IP deletion in mice results in hypertension and reduced anticoagulation activity [114].

In human blood, PGI_2_, measured as PGF_1*α*_, is 400 pg/mL in new born infants, 230 pg/mL in infants, 150 pg/mL in adolescents, and 85 pg/mL in adults [112]. Age-associated PGI_2_ decline is also observed in urine of humans [115, 116]. The endothelium is the main site for PGI_2_ synthesis [50, 51]. Although there has been no report on PGI_2_ production in isolated human vessels, PGI_2_ levels were reported to decline in cultured human vascular endothelial cells during serial passage [117–119]. Based on these reports, one would expect that PGIS in endothelium decreases with age. Yet there have been no reports evaluating age-associated PGIS changes in the human endothelium. In the endothelial cells from rat aorta, there is a slight and insignificant age-associated decrease in PGIS mRNA [84]. However, additional evidence shows that mRNA or protein of PGIS is 2–4-fold higher in aorta or coronary arteries of aged normotensive rats [85, 86, 110, 120] suggesting that lower PGI levels may be caused by increased PGI_2_ degradation with age, rather than the change in PGI_2_ synthesis. In fact, there is no apparent correlation between circulating PGI_2_ level with level of endothelial PGIS, suggesting the necessity of investigation of the effects of age on the metabolism/degradation of PGI_2_. More work is needed to determine whether circulating PGI_2_ correlates to endothelial PGI_2_ and to clarify the effects of age on PGI_2_ in the endothelium and in the circulation. Age-associated reduction in IP level has been consistently reported in rats [84, 85]. The reduced IP is expected to lead to reduced sensitivity to PGI_2_ effects. Consistently, dilation in response to PGI_2_ is significantly blunted in aged humans as determined by forearm blood flow measurements [121].

Reports on the change in PGI_2_ or PGIS under pathological conditions, such as hypertension, are contradictory. While one group reported a 50% reduction in PGI_2_ in SHR aorta as compared to WKY aorta [96], another group reported insignificant differences in PGI_2_ levels in SHR and WYK rats [64, 65]. In addition, Tang and Vanhoutte reported that PGI_2_ mRNA is 4-fold higher in the endothelial cells of SHR aorta than in WKY aorta [84]. These limited and inconsistent reports indicate a need for more complete and thorough investigations into how aging affects PGI_2_, its synthase, receptor, and metabolism. Moreover, clarifying PGI_2_ effects in the development of cardiovascular disorders in animal models and in humans could be of potential therapeutic significance.

## 4. PGE_2_

Prostaglandin E_2_ (PGE_2_) is the most abundant prostaglandin in the human body. In endothelium, however, its level is lower than that of PGI_2_, in line with a lower expression level of the corresponding synthases, which are 5–100-fold lower than PGIS [54, 64, 65, 84]. There are three types of known PGE_2_ synthases (PGESs), the cytosolic PGES (cPGES) and two forms of membrane PGES, mPGES1 and mPGES2 [122, Figure 1]. cPGES is constitutively expressed and functionally coupled to COX1 [122, 123]. mPGES1 is inducible and functionally coupled with COX2 [124] and is the major PGE_2_ synthase responsible for PGE_2_ production [123]. In endothelium, the expression levels of the PGESs are comparable to other PG synthases [54, 64, 65, 84]. Consistently, the amount of PGE_2_ in endothelium is comparable to other PGs, but lower than the amount of PGI_2_ [54, 97, 107–110, 125]. In further accord, the contribution of PGE_2_ to endothelium-dependent vasoaction is marginal [84, 125]. Chen et al. showed that deletion of mPGES1 in mice resulted in abolished production of PGE_2_ but did not affect blood pressure [114]. Yang, on the other hand, showed that mPGES1 deletion in mice resulted in exaggerated hypertensive in response to high salt and angiotensin II infusion [126], suggesting that mPGES1 may be an important physiological regulator of blood pressure. While the role of mPGES1 in blood pressure regulation is debatable, mPGES1 is implicated in atherosclerosis. Deletion of mPGES1 in mice retards atherosclerosis development [127].

PGE_2_ acts through four PGE_2_ receptors (EP1, EP2, EP3, and EP4), which are mainly located in the smooth muscle cells in the vessels [125, 128]. Activation of EP1 and EP3 receptors induces calcium mobilization/release and inhibits adenylyl cyclase release, which triggers vasoconstrictions [111, 129]. In contrast, activation of EP2 and EP4 receptors stimulates adenylyl cyclase and induces cyclic adenosine monophosphate release, which triggers vasorelaxation [111, 129]. The vascular actions of PGE_2_ are complex due to the opposing vasoactions triggered by the binding of PGE_2_ to the variant PGE_2_ receptors. Depending on the circumstances, PGE_2_ may be vasodilating [47, 67–70] or vasoconstricting [53, 54, 71–73]. In addition to the distributions of different PGE_2_ receptors expressed in the vascular system, PGE_2_ concentration is also important. This complexity likely explains the reported inconsistent effects of mPGES1 deletion on blood pressure [114, 126]. PGE_2_ has a biphasic effect on human blood platelet aggregation. At low concentrations (0.01–1 *μ*M), it potentiates platelet aggregation, and, at higher concentrations (10 *μ*M), it inhibits ADP- and collagen-induced aggregation in platelet rich plasma [71, 130–132]. The endogenous PGE_2_ concentration is below 1 *μ*M [133], making PGE_2_ a stimulator of atherosclerosis. Thus, reduced PGE_2_ level by mPGES1 deletion retards atherosclerosis development [127].

There is little information available on age-related changes in any of the PGESs, PGE_2_, or EPs. A recent report by Tang and Vanhoutte revealed that while cPGES and mPGES1 in the aorta endothelial cells are insignificantly higher in aged rats, mRNA of mPGES2 is 5-fold higher [84], which can presumably result in higher level of PGE_2_. PGE_2_ secreted from coronary arteries is increased in aged rat as compared to young rats [120]. Expression of EP1–4 increased with age, with EP4 elevated 2-fold in endothelial cells from rats of 72 weeks as compared with rats of 36 weeks [84]. Since vasoaction depends on the ligand and the type of receptors, age-increased PGE_2_ and EP4 are assumed to predispose to increased vasodilation. Further investigation is required to determine the effect of age-related changes in PGE_2_ and its synthases and receptors in different vascular beds and on relaxation/constriction of vasculatures.

## 5. PGF_2*α*_

There are two isomers of prostaglandin F_2*α*_. One is PGF_2*α*_, and the other is 9*α*, 11*β*-PGF_2_ [134–137]. They are transformed from PGH_2_ by the membrane-associated 9,11-endoperoxide reductase and from PGD_2_/PGE_2_ by cytosolic PGD_2_ 11-ketoreductase/PGE_2_ 9-ketoreductase, respectively [138, Figure 1]. In endothelium, the level of PGF_2*α*_ is similar to that of PGE_2_, but much lower than that of PGI_2_ [54, 97, 107–110, 125], corresponding to low abundance of PGF_2*α*_ cognate synthase (PGFS) in the endothelium [54, 64, 65, 84].

PGF_2*α*_ has its own specific receptor (FP), which is expressed in endothelium and in vascular smooth muscle cells [139–143]. PGF_2*α*_ can also interact with TP [54]. Interaction between PGF_2*α*_ and its receptor generates calcium release and triggers potent vasoconstriction [144–148]. Deletion of FP reduces arterial blood pressure and delays atherogenesis in hyperlipidemic mice [149]. PGF_2*α*_ has also been indicated in promoting cardiac hypertrophy [150–152]. Although PGF_2*α*_ is a potent vasoconstrictor, the contribution of PGF_2*α*_ to endothelium-dependent contractions is minimal in most cases due to its relatively low abundance in the endothelium [54, 97, 107–110, 125].

Information on the effects of aging on PGF_2*α*_ is limited. PGFS mRNA was doubled in the endothelial cells from aged rat aorta as compared to that from young rat aorta [84]. Consistently, PGF_2*α*_ is 2-fold higher in the aorta of aged rats versus young rats [110, 148]. Change in FP mRNA in the endothelial cells of rat aorta with age, however, is insignificant [84]. Basal PGF_2*α*_ is slightly higher in the aorta of SHRs than that of WKY rats, but the difference is increased upon acetylcholine stimulation [54]. Research needs to be conducted to obtain more complete information on age-associated changes in PGF_2*α*_ in humans and the effects of those changes on the development of cardiovascular disorders.

## 6. PGD_2_

PGD_2_ is synthesized by two PGD_2_ synthases (PGDSs) encoded by two unrelated genes. One is hematopoietic PGDS (H-PGDS), and the other is lipocalin-type enzyme (L-PGDS) [138, Figure 1]. Both can be upregulated in response to an increase in fluid shear stress [153]. In most of the vasculatures, the level of PGD_2_ is very low or undetectable in some vascular beds [74], due to the low level of PGDSs [54, 64, 65, 84].

PGD_2_ has multiple receptors [154]. However, two PGD_2_ receptors (DP1 and DP2) have been most widely studied (Figure 1). Besides playing an important role in the central nervous and immune systems [154], PGD_2_ has functions in the vasculature. PGD_2_ can elicit endothelium-dependent relaxation through receptor activation [59] and acts as a vasodilator [155, 156]. On the other hand, it can also act as a bronchoconstrictor [157–159]. Finally, PGD_2_ is an anticoagulant [160–163].

There is only one report on the effect of aging on PGDS and DP. While aging had no effect on L-PGDS, it caused a 5-fold increase in H-PGDS mRNA in aged rat aorta endothelial cells [84]. Age had no apparent effect on DP [84]. H-PGDS is 3-fold higher in aorta endothelial cells from SHRs versus WKY rats, whereas L-PGDS is decreased in these cells in SHRs versus WKY rats [84]. In the smooth muscle cells from the same aorta preparations, DP mRNA was measured to be 3-fold higher in SHRs as compared with WKY rats [84].

## 7. TxA_2_

TxA_2_ is mainly produced in the platelets [100, 101]. It is also synthesized in the vasculature, the endothelium, and smooth muscles by TxA_2_ synthase (TXS) [49, Figure 1]. However, the amount of TxA_2_ in the endothelium is much lower than the amount of PGI_2_ [54, 97, 107–110, 125]. Consistently, the expression level of the TXS is much lower than that of PGIS [54, 64, 65, 84].

There are two types of TxA_2_ receptors (TP) denoted, TPa and TPb. TP interacts with TxA_2_ and other PGs, although TxA_2_ is the most potent agonist [54, 164]. TP appears to be the main receptor of PGH_2_ [25, 26, 90–97]. Deletion of TP receptors has provided insights into their physiological function. For example, TP knochout mice exhibit decreased vascular proliferation and platelet activation in response to intimal lesions [165]. These animals also experience delays in atherogenesis [166]. TP deletion also prevents angiotensin-II- and L-NAME-induced hypertension and associated cardiac hypertrophy [167].

TxA_2_ elicits diverse physiological/pathophysiological reactions, including platelet aggregation and vascular smooth muscle contraction [49]. Activation of platelet aggregation is thought to be the dominant biological function of TxA_2_. TxA_2_ causes platelet shape change, aggregation, and secretion, which promotes thrombus formation and thrombosis [168–171]. Thrombosis can cause acute myocardial infarction and atherogenesis [166, 171–174]. TxA_2_-induced contraction effects are variable, depending on the specific vascular beds examined and the agent used to induce contraction [116, 175, 176]. The majority of reports coincide with the view that the contraction induced by endothelium-derived TxA_2_ is weak, because inhibitors of TXS do not induce relaxation [91, 92, 96, 176]. Contraction effects are likely mediated by TP activated by PGH_2_ because inhibitors of PGHSs and TP induce relaxation [91, 92, 96, 175, 176]. 

Several publications reported a 2–5-fold increase in TxA_2_ in aorta or mesenteric arteries of aged rats as compared to that of young rats [42, 86, 172]. Consistently, Tang and Vanhoutte reported a 4-fold increase in TXS mRNA [84]. In contrast, a single investigation of age-dependence of TxA_2_ did not find any significant difference in TxA_2_ between young and aged rat aortas [110]. Aging did not show any significant effect on rat aorta TP mRNA [84].

An increased production of TxA_2_ has been found in patients and animal models of several cardiovascular diseases including unstable angina [177], experimental myocardial ischemia and infarction [178], cerebral vasospasm, pregnancy induced hypertension [179, 180], and congenital heart disease [116]. TxA_2_ levels reported in those studies are systemic, rather than endothelial. In endothelium, there is no difference in aorta TxA_2_ between SHRs and WKY rats [54, 64, 65, 87]. However, TXS mRNA is doubled in the aorta endothelium of SHRs versus WKY rats [84]. Age-related changes in TP have not been found [84, 181].

**Figure 2:**
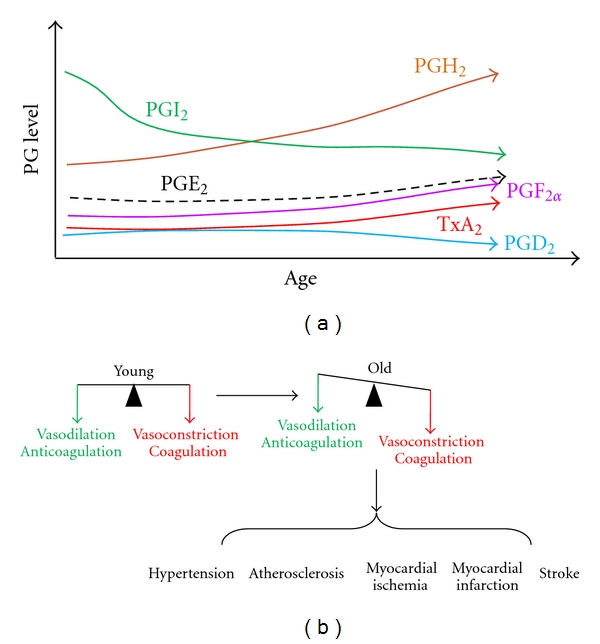
Age-shifted PG profile (a) and vasoaction (b). (a) As age advances, most of the PGs and TxA_2_ increase, whereas PGI_2_ decreases. (b) The age shifts PG profile toward vasoconstriction and coagulation causing several cardiovascular disorders.

In summary (Table 1), aging has been consistently shown to cause severalfold increase in COXs, that is, the synthesis of PGH_2_ [29, 42, 63, 84–86]. Aging probably reduces PGI_2_, the predominant PG in the endothelium [112, 115–118, 182], though it is not certain and requires more work. Aging has been shown, or has the potential, to change other PGs in the endothelium. However, because the level of PGI_2_ is 10–100-fold higher than that of the rest of PGs, the shift of PG profile in the endothelium during aging will be predominantly determined by PGI_2_ and untransformed PGH_2_. PGI_2_ and PGH_2_ have opposing effects on vessels and platelets. The net result of the effects of aging will be a shift toward a proconstrictive mediator profile, as shown in Figure 2.

## 8. Association of Prostaglandin and Cardiovascular Disorders in Aging

Associated with this shift are several cardiovascular disorders including hypertension, atherosclerosis, myocardial ischemia, myocardial infarction, and stroke (Figure 2(b)). Reduced ratio of PGI_2_/TxA_2_ was observed in elderly hypertensive patients [183–186]. Age impaired PGI_2_ synthesis [84, 187] is associated with hypertension [84], progression of atherosclerotic lesions [188], and increased thrombotic risk and heart failure [189, 190]. In addition, aging not only reduces the expression of IP [84], but also reduces the sensitivity of IP [182, 191]. These factors might contribute to the progression of atherosclerosis, as mice with deleted IP [192, 193] and human patients with a dysfunctional prostacyclin IP receptor mutation [194] show accelerated atherothrombosis [97].

 On the other hand, aging induces TXS [84]. Higher concentrations of TxA_2_ are observed in serum or urine in several age-related and hypertensive diseases [185, 186, 195]. In the atherosclerotic coronary artery, the density of TP receptor is increased [171]. Aging-increased TxA_2_, together with induced TP in the atherosclerotic coronary artery, accelerates arterial atherosclerosis, leading to myocardial infarction [191]. The TP-mediated signaling can also be triggered by PGH_2_. Age increases COX1/2 in animals and human [21, 29, 33, 34, 42, 63, 84–86] and thereby increases PGH_2_ production. Age-increased expression of COX-2 in coronary, carotid, and femoral arteries is associated with human atherosclerosis [196–199].

## 9. Therapeutics That Modulate Prostaglandins in Cardiovascular Disorders

Because prostaglandins and thromboxane are such important factors in endothelium functions and therefore in the physiology and pathology of the vascular system, numerous pharmacological agents that target these factors have been developed to mitigate cardiovascular diseases. As listed in Table 2, prostacyclin (PGI_2_) and analogues are used clinically to treat hypertension, especially pulmonary hypertension [75, 200–202]. They are also used to inhibit arterial thrombosis and ameliorate myocardial ischemia [203–207]. Although the vascular actions of PGE_2_ are complex, PGE_2_ and analogues are used to reduce blood pressure and to alleviate congestive heart failure [208–210], owing to their ability to stimulate renin release and natriuresis and diuresis [211–213]. PGE_2_, PGE_1_, and their analogues are more often used to maintain the patency of the ductus arteriosus in infants with congenital heart disease [214–217]. Antagonists of TXS and TP are potent antithrombosis agents and used to treat atherosclerosis, myocardial ischemia, and stroke [218–227].

The underlying principle of the design of these drugs is to selectively increase the effects of vasodilators and anticoagulators and to selectively reduce the effects of vasoconstrictors and coagulators by modulating the amount of ligands, synthases, or receptors of a specific eicosanoid. Because prostaglandins and thromboxane A_2_ are from the same precursor but elicit opposing effects, selectivity is crucial in the design of these therapeutics. Nonselective inhibition of the upstream synthases, COX1 and COX2, can result in undesirable side effects including hypertension, manifestation of myocardial ischemia, and increased incidents of acute myocardial infarction and stroke, which occur more often in the elderly [104, 228–230].

Intriguingly, low dose of aspirin, an inhibitor of COX1, is popularly used in the prevention of cardiovascular diseases [231–233]. Aspirin covalently acetylates a specific serine moiety (serine 530 of COX-1 and serine 516 of COX-2) [234, 235], and its binding to COX1 is about 170-fold stronger than that to COX-2 [236]. Thus, aspirin is a covalent inhibitor of COX1 inactivating it irreversibly. TxA_2_ is mainly produced in platelets [100, 101], whereas PGI_2_ is mainly produced by endothelial cells [51, 57]. Different from most other cell types, platelets do not possess nuclei, which are required for protein synthesis. While COX1 can be regenerated in other cells, such as endothelial cells, COX1 cannot be regenerated in platelets. Nor can COX1 activity be recovered after inactivation by aspirin. Therefore, low dose of aspirin irreversibly and selectively inhibits TxA_2_ production in platelets.

However, new platelets are constantly formed, and TxA_2_ is persistently produced [237], which leads to a need for continuous dosing to constantly inhibit COX1. Aspirin resistance is a common clinical phenomenon [238] and has been observed for more than twenty five years [239]. Aspirin resistant patients, partially due to inherited polymorphisms in COX1 [240, 241], have a nearly 4-fold increase in risk of suffering a vascular event compared with aspirin responders [242–244]. As an alternative to aspirin therapy, antagonists of TXS and TP, which can also be combined with aspirin, have been applied to ameliorate thrombosis and prevent cardiovascular diseases [226].

## 10. Conclusion and Perspective

The incidence and prevalence of cardiovascular diseases increase with advancing age, to the extent that age has been identified as the dominant risk factor for these pathologies [2, 4–6]. It is well established that PGs are powerful endogenous vasodilators and vasoconstrictors and platelet aggregators, playing important roles in regulating homeostasis in vascular systems. Although limited, the current analysis of the literature suggests that there is a modified PG profile associated with age and indicates that age has significant effects on the abundance of PGs, their synthesis, as well as their signaling transduction pathways. Aging-modulated PG profile offers a potentially important molecular mechanism underlying age-dependent endothelial dysfunction and age-associated cardiovascular diseases. Knowledge of age-associated PGs profile changes can be important for designing new pharmacological interventions to prevent or slow down age-associated cardiovascular diseases. Given their biological roles, improved investigation of age-associated changes in PG synthesis, metabolism, and signaling in all major vascular beds is needed.

It is clearly difficult to obtain human vascular tissues to determine age associated changes. Surrogate tissues and fluids such as human blood or urine are plentiful but are of limited value for assessing tissue-specific effects. Defining the relationship between PGs, particularly PGI_2_ and PGH_2_, in vascular tissues and the amounts in blood or urine in animal models could be helpful to interpret PG profiles in humans. Technical challenges exist due to metabolite instability. For example, PGH_2_ is transformed to other PGs and is biologically important in its own right, but untransformed PGH_2_ is difficult to measure [98, 245]. The development of user-friendly methods could facilitate acquiring these measurements [91, 98, 245]. For example, PGH_2_ can be instantly reduced to 12-heptadecatrienoic acid (12-HHT) by FeCl_2_ [91, 98, 245]. 12-HHT is stable and inactive and measurable [91, 98, 245]. Therefore, total PGH_2_ can be measured as 12-HHT. A relatively mild reducing agent, SnCl_2_, can reduce untransformed PGH_2_ to PGF_2*α*_. Untransformed PGH_2_ can be calculated by subtracting the estimate of PGF_2*α*_ in samples without SnCl_2_ from the corresponding estimate in samples with SnCl_2_ [91, 98, 125]. Alternatively, epidemiological approaches could avoid these technical difficulties and offer valuable genetic information. Haplotype analyses have revealed that several polymorphisms in COX, PGIS, and IP are associated with age and cardiovascular diseases [246–250].

Research on an important aspect of age-associated changes in PGs is largely absent in the literature; that of age-associated effects on PG metabolism. One of the most important features of PGs is rapid clearance. Most PGs are metabolized to inactive forms within 1–3 minutes [119, 251], and consequently their signaling is terminated within that time frame. This is due to an effective and efficient metabolism system mainly composed of prostaglandin transporter (PGT) and 15-hydroxyprostaglandin dehydrogenase (15-PGDH) [252]. Both PGT and 15-PGDH have been shown to regulate PG degradation [245, 253, 254]. Thus far, there have been no reports on the influence of age on PG metabolism.

In conclusion, PGs and TxA_2_ play critical roles in many important events involved in the normal functions of vascular system, including vasodilation, vasoconstriction, platelet aggregation, and inflammation. Although these eicosanoids were discovered in the 1970s, the research into age-associated shifts of the PG profile has just begun. Age-associated alterations in PG profiles are not only interesting, but also important in defining the molecular mechanisms of age-associated cardiovascular pathological conditions and informing strategic and personalized prevention and cure of those diseases.

## Figures and Tables

**Table 1:** Age-associated changes in PGs and TxA_2_ and their synthases and receptors.

Entity	Tissue	Age	Change	References
COX1/2 (hum, r, m)	Mesenteric microvessels	Adult, aged	Increase	[21, 29, 33, 34, 42, 63, 84–86]
PGI_2_ (hum)	Blood	Adolescent, aged	Decrease	[112, 116]
PGIS (r)	Aorta, coronary artery in heart	Adults, aged	Increase	[85, 86, 110, 120]
IP (r)	Aorta	Adults, aged	Decrease	[84, 85, 121]
PGE_2_ (r)	Coronary artery in heart	Aged	Increase	[120]
cPGES (r),	Aorta	Old adult	N/S	[84]
mPGES-1 (r)	Aorta	Old adult	N/S	[84]
mPGES-2 (r)	Aorta	Old adult	Increase	[84]
EP1–3 (r)	Aorta	Old adult	N/S	[84]
EP4 (r)	Aorta	Old adult	Increase	[84]
PGF_2*α*_ (ham, r)	Aorta	Aged	Increase	[110, 148]
PGFS (r)	Aorta	Old adult	Increase	[84]
FP (r)	Aorta	Old adult	N/S	[84]
PGDS (r)	Aorta	Old adult	Increase	[84]
DP (r)	Aorta	Old adult	N/S	[84]
TxA_2_ (r)	Aorta or mesenteric artery		Increase	[42, 86, 172]
TXS (r)	Aorta	Old adult	Increase	[84]
TP (r)	Aorta	Old adult	N/S	[84]

hum: human; ham: hamster; r: rat; m: mouse; N/S: not significant.

Definition of age groups: human, adolescent, 13–19 years; adult, 20–60 years; aged, >60 years. Hamster, aged, >18 months. Rat, young adult, 3–6 months; old adult, 6–18 months; aged >24 months.

**Table 2:** Prostaglandin-related pharmacological agents in the treatment of cardiovascular diseases.

Modulator	Drugs (trade name)	Clinical application	References
PGI_2_ and its stable analogues	Epoprostenol sodium (Flolan), Beraprost sodium (Procyclin), Iloprost (Ventavis), Treprostinil (Remodulin)	Primary pulmonary hypertension, pulmonary arterial hypertension	[200–207]
PGE_2_ and its analogues	Dinoprostone, Viprostol	Congenital heart disease	[208–210, 214–217]
TXS inhibitors	Dazoxiben, Camonagrel, Picotamide (Dusodril)	Thrombosis, atherosclerosis, arrhythmias	[218–226]
TP inhibitors	Picotamide, S18886 (Triplion)	Thrombosis, atherosclerosis, ischemic stroke, myocardial infarction	[84, 223–227]
COX1 inhibitor	Aspirin	Thrombosis, atherosclerosis, ischemic stroke, myocardial infarction	[231–235]
